# The Effects of Postpartum Yak Metabolism on Reproductive System Recovery

**DOI:** 10.3390/metabo12111113

**Published:** 2022-11-15

**Authors:** Shi Shu, Changqi Fu, Guowen Wang, Wei Peng

**Affiliations:** Academy of Animal Science and Veterinary Medicine, Qinghai University, Xining 810016, China

**Keywords:** liquid chromatography–mass spectrometry, yak, perinatal period, metabolomics, reproduction

## Abstract

The goal of this study was to determine the metabolism of multiparous female yaks during the late perinatal period and identify its effects on reproductive recovery in order to explain the low reproduction rate of yaks. Eight multiparous female yaks were randomly selected as the sample, and serum was collected from the yaks every 7 days from the day of delivery until 28 days after the delivery (five time points). The presence of serum metabolic profiles and reproductive hormones was identified using ELISA. The key metabolites were identified using liquid chromatography–mass spectrometry, and a dynamic metabolic network representation was created using bioinformatics analysis. A total of 117 different metabolites were identified by calculating the fold change of the metabolite expression at each time point. The dynamic metabolic network was created to represent the activities of the key metabolites, metabolic indexes and reproductive hormones. The initial efficiency of the glucose metabolism in the late perinatal period was found to be low, but it increased during the final period. The initial efficiencies of the lipid and amino acid metabolisms were high but decreased during the final period. We inferred that there was a postpartum negative energy balance in female yaks and that the synthesis and secretion of estrogen were blocked due to an excessive fatty acid mobilization. As a result, the reproductive hormone synthesis and secretion were maintained at a low level in the late perinatal period, and this was the main reason for the delayed recovery of the reproductive function postpartum. However, the specific mechanism needs to be further verified.

## 1. Introduction

The yak (*Bos grunniens*) is a mammal that lives at high altitudes. It is an important plateau animal that provides many human necessities [1,2]. However, yaks have a low reproduction rate [3,4]. The long calving intervals limits the reproductive rate of yaks [5,6]; estrus usually occurs from July to October of each year and is followed by calving from April to June in the following year [7]. Many yaks have only one annual estrus whether they calve in that year or not, and the next estrus usually does not occur until the following year. Yaks are therefore most likely to calve once every 2 years or twice in 3 years [8,9]. The most effective way to increase the reproductive rate of a yak is to restore estrus, and the subsequent reproduction, within 3–4 months after the delivery and so to ensure pregnancy in the same year as parturition. It has been found that the postpartum recovery of yaks depends mainly on the environmental conditions and their nutrition in winter and spring [10,11], as well as lactation [12,13]. Early weaning promotes a reproductive recovery in yaks and subsequent estrus when compared with a calf’s nurture by lactation for 1–2 years [14,15]. The 3–4-month postpartum recovery period includes a very special stage, the late perinatal period (LPP), that lasts for about four weeks. Uterine inversion in yaks is completed within about 30 days after calving. During the LPP, the hypothalamus–pituitary–ovary axis synthesizes the luteinizing hormone (LH) in the anterior pituitary, and the ovaries respond to the LH pulse spike and estrus commences [16,17]. In fact, the state of most female mammals during the LPP can affect the recovery of their reproductive system. However, the research on yaks is almost blank. In the study of dairy cows, it was found that the physical condition during the LPP can directly affect the postpartum estrus cycle, pregnancy rate and embryo survival rate [18,19]. The occurrence of diseases during the LPP can directly delay the recovery of postpartum estrus in dairy cows and increase the risk of pregnancy loss [20]. Feeding management in the LPP is mostly absent as a topic in yak research and there is little available data for the feeding and metabolism of yaks during the LPP.

We conducted a metabolomic study to gather data and increase our understanding of the dynamic metabolic changes in female yaks during the LPP. In a 2019 metabolomic study, Li and Jang [21] identified metabolic changes in yak mammary glands. Liquid chromatography–mass spectrometry (LC–MS) is a recently developed technique used in metabolomics to detect a small molecular metabolite presence in biological samples [22]. LC–MS can detect a large number of complete metabolites and analyze compounds without any prior knowledge of their composition [23,24]. LC–MS has been used to analyze metabolites in the yak rumen [22]. It has also been used to produce metabolic profiles of milk, dairy cow blood and bull sperm [25,26,27]. LC–MS has also been used in reproductive research to detect metabolic changes in progesterone in cattle during estrus [28].

The purpose of this study was to provide the basic metabolic data of yaks during the LPP using LC–MS. Our analysis provides basic information for the feeding and management of yaks in the LPP and provides a theoretical justification for reducing the calving interval and thus increasing the reproductive rate of yaks.

## 2. Method and Materials

### 2.1. Experimental Design and Sample Collection

The experimental animals (*n* = 25) were selected from multiparous female yaks in the LPP in Haiyan County of Qinghai province. There was no signification difference in age, parity or body condition among the yaks (Supplement File Table S1). Each experimental yak had previously given birth normally, without dystocia, stillbirth or fetal casing, to healthy calves. The yaks foraged in open grassland during the day and returned to a static enclosure at night. The calves followed their mothers from 3 to 5 days after birth and drank maternal milk at will. Blood samples were collected at birth and every 7 days from calving until day 28 postpartum. Experienced veterinarians checked the specimen yaks for common diseases and reproductive diseases every time blood was collected. Diseased yaks were excluded from the study. The milestone time points for the blood samples were A (calving day, day 0), B (day 7 postpartum), C (day 14 postpartum), D (day 21 postpartum) and E (day 28 postpartum). The blood was collected without any anticoagulant. The serum was collected after the blood had stood at room temperature (approximately 20 °C) for 3 h. The blood was centrifuged at 3000 rpm for 5 min, and the extracted supernatant was stored at −80 °C. Animal care and use were performed according to Animal Management Regulations (Ministry of Science and Technology, China, revised in March 2017) and were approved by the Institutional Animal Care and Use Committee, Qinghai University, Xining, Qinghai, China (2022-QHMKY-016).

### 2.2. Detection of Main Substances in Serum

We conducted tests of the serum of female yaks during the LPP to identify the principal metabolites. We detected non-esterified fatty acids (NEFA), triglycerides (TG), β-hydroxybutyric acid (BHBA), glucose (GLU), total protein (TP), estradiol (E_2_), progesterone (P4), the follicle stimulating hormone (FSH) and the luteinizing hormone (LH) in our examination of the lipid metabolism, glucose metabolism, protein metabolism and reproductive hormone secretion. All items except GLU were tested with an ELISA kit (Shanghai Rongsheng Biotech Co., Ltd., Shanghai, China, 1023-01-01-07), which uses a glucose oxidase method. ELISA kits were purchased from Jiangsu Meibiao Biotechnology Co., Ltd., Nanjing, China, (BHBA: MB-9690A; NEFA: MB-5201A; TG: MB-4922A; TP: MB-9693A; E_2_: MB-2163A; P4: MB-4772A; FSH: MB-5413A; LH: MB-5289A); detection was by a multimode reader (Biotek, SYNERGY2). The tests were conducted according to the manufacturer’s instructions, as in previous studies [29].

### 2.3. Metabolite Profiling Analysis

We selected 8 of 25 yaks for the metabolomic experiments (the information in Supplement File Table S1). A 100 μL serum sample was mixed with 300 μL of lysis buffer (80% methanol). The sample was uniformly mixed using a Vortex mixer (Haimin Kylin-Bell Lab Instruments Co., Ltd., Haimen, China, QL-901) after standing at −20 °C for 30 min and then centrifuged at 14,000 rpm for 30 min. The supernatant was extracted and filtered using a 0.45 μm filter membrane. A measure of 20 μL of supernatant was transferred to an injection bottle (Thermo Scientific, Wilmington, DE, USA, 00280027) and prepared for LC–MS analysis. Equal amounts (20 µL) of the supernatant were taken from all samples and mixed to become the quality control (QC) samples. High performance liquid chromatography (Thermo Scientific Dionex ΜultiMate 3000 Rapid Separation LC (RSLC)) and mass spectrometry (Thermo Scientific Q Exactive) were performed. The chromatographic separation was performed using a Waters ACQUITY UPLC BEH C8 (2.1 × 100 mm, 1.7 μm column). The ion source was HESI, the spray voltage was 2500 V and the capillary temperature was 320 °C. The mobile phase A solvent was acetonitrile (ACN)/H2O (60/40), and the B solvent was isopropyl alcohol/ACN (90/10); 0.1% formic acid and 10 mmol/L ammonium formate were in both mobile phases. The samples were eluted with a linear gradient, and the loading pump was set at 0.25 mL/min. During the 20 min, the gradient changed in the first 5 min from 2% A and 98% B to 70% A and 30% B; in min 8, the gradient changed to 100% A and 0% B until min 16, when the gradient began to return to the initial conditions. Thermo Q Exactive was operated in positive and negative modes at a mass resolution of 70,000 with a m/z range of 100–1500. A QC sample was used to check the signal reproducibility after every 8 samples.

### 2.4. Raw Data Analysis and Multivariate Statistical Analysis

The LC–MS raw data in uep format were imported into Progenesis QI 2.3 (PQI) software. The peak alignment was carried out with a retention time (rt) deviation of 0.2 min and a mass deviation of 5 ppm. The peaks were detected with a coefficient of variance (CV) of 30%, a signal to noise ratio of 3 and a minimum signal strength of 100,000. The molecular formula was predicted by a molecular ion peak and fragmented ions and was compared with the human metabolome database (HMDB, http://www.hmdb.ca/, accessed on 20 March 2022) to obtain the qualitative results of signal peaks. HMDB contains 114 026 metabolic records (water-soluble and fat-soluble metabolites) and links to discoveries in metabolomics, clinical chemistry, biomarkers and other fields. The results obtained also include metabolite IDs from the KEGG (Kyoto encyclopedia of genes and genomes) pathway database.

Multivariate statistical analysis primarily used Simca-P software (version 13.0, http://www.umetrics.com/simca, accessed on 20 March 2022) for principal component analysis (PCA), partial least squares-discriminant analysis (PLS-DA) and orthogonal partial least squares-discriminant analysis (OPLS-DA). The identification of the differential metabolites was pairwise between groups at different time points (B vs. A, C vs. A, C vs. B, D vs. A, D vs. B, D vs. C, E vs. A, E vs. B, E vs. C and E vs. D). Differential metabolites were identified by a fold change (FC, FC > 2 or FC < 0.5), a variable importance in the projection (VIP, VIP > 1) and the *p* values (*p* < 0.05) of the *t*-tests between the pairs of groups. The VIP score was calculated using PLS-DA. The differences between the main compounds in the serum were analyzed using a one-way ANOVA (IBM SPSS software version 26.0). A pathway analysis was performed using the KEGG database (https://www.genome.jp/kegg/, accessed on 20 March 2022).

### 2.5. Bioinformatics Analysis of Differential Metabolites

The KEGG (https://www.genome.jp/kegg/, accessed on 20 March 2022) functional annotation, HMDB annotation and LIPID MAPS structure database (LMSD, http://www.lipidmaps.org/data/structure/, accessed on 20 March 2022) annotation were used to analyze the screened differential metabolites. Combining these three databases enabled a better determination of the metabolic changes of pregnant female yaks during the LPP.

## 3. Results

### 3.1. Change Trends of Major Serum Compounds during LPP

**Figure 1:**
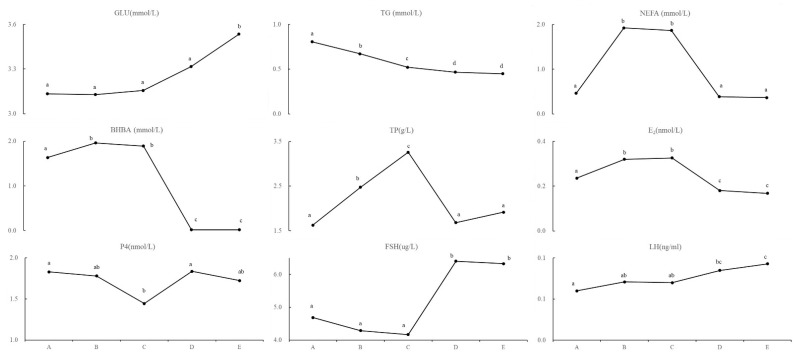
The detective results of serum indexes. GLU: glucose; TG: glucose; NEFA: non esterified fatty acids; BHBA: β hydroxybutyric acid; TP: total protein; E_2_: estradiol; P4: progesterone; FSH: follicle stimulating hormone; LH: luteinizing hormone; A–E: every 7 days from the day of delivery until 28 days after delivery (5 time points). There was significant difference between each two time points marked different letter (*p* < 0.05).

The detection of the principal compounds in yak serum allowed us to identify change trends over time (Figure 1) and the details are shown in the information in Supplement File Table S2. The GLU concentration showed an increasing trend and was significantly different at time E from the GLU concentrations at other time points (*p* < 0.05). This suggests that yak calving resulted in the consumption of large amounts of glucose until the end of the perinatal period. The NEFA and BHBA trended similarly to each other, first increasing (at times A, B and C) and then decreasing (at times D and E), and the differences between them were significant (*p* < 0.05). This indicates that the mobilization of fatty acids and the production of ketone bodies begins in yaks after a postpartum energy loss. This effect was also indicated by the significant continuous decrease in the TG (*p* < 0.05). The continuous increase in the TP at times B and C was significant (*p* < 0.05). We infer that this was due to the compensatory energy supply for the protein loss.

When we examined the reproductive hormones, we found that the trend of E_2_ was consistent with that of the BHBA, which suggests that the secretion of E_2_ is related to the mobilization of fatty acids. P4 showed a significant temporary decrease at time C when compared with other time points (*p* < 0.05). Both the FSH and LH showed an upward trend until the FSH showed a significant rapid increase at times D and E (*p* < 0.05), while LH showed a continuous upward trend with a nonsignificant difference between the two adjacent time points D and E (*p* > 0.05).

### 3.2. Profile of Yak Metabolism during LPP

**Figure 2:**
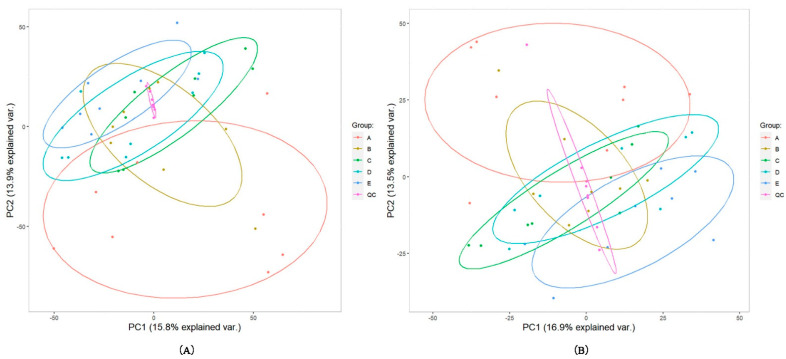
The PCA analysis. A–E (in figure): every 7 days from the day of delivery until 28 days after delivery (5 time points). QC: quality control (all samples mixed). The figure (**A**) and (**B**) was positive and negative ion mode, respectively.

**Figure 3:**
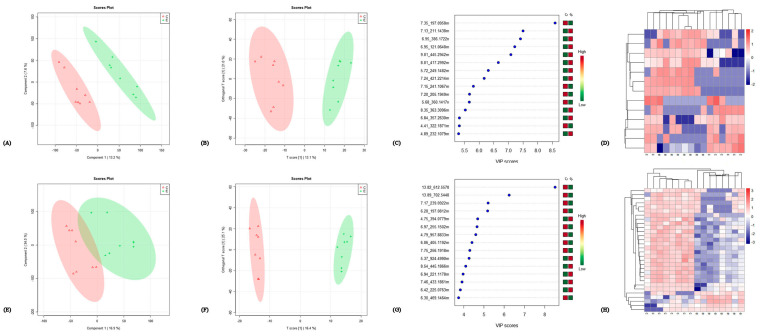
PLS-DA, OPLS-DA, VIP scores and cluster diagram analysis results taking E/C as an example. The figures in the first line (**A**–**D**) are the analysis results in the positive ion mode, which are, respectively, PLS-DA, OPLS-DA, VIP scores and cluster diagram analysis; the second line (**E**–**H**) are the analysis results in the negative ion mode.

In total, 2841 of the 5801 detected compound signals were qualitatively analyzed in a positive ion mode. In a negative ion mode, 1326 of 2813 compounds were qualitatively analyzed. The correlation analysis of the QC samples in positive and negative ion modes showed that the correlation was very high in the positive ion mode, with correlation coefficients >0.99 (Supplement File Figure S1). The PCA was undertaken for all the compound signals (Figure 2) in positive and negative ion modes. The QC samples were close to each other and were highly aggregated and stable. The samples from other groups were also aggregated with each other. In a positive ion mode, the proportions of variance explained by variables PC1 and PC2 were, respectively, 15.8% and 13.9% (Figure 2A). The corresponding values in the negative ion mode were 16.9% and 13.5% (Figure 2B). We performed PCA, PLS-DA and OPLS-DA on the data for each pair of time points. Figure 3 shows the VIP scores of the highest ten compounds given by PLS-DA and OPLS-DA and the heat map of differential metabolites of E/C in positive and negative ion modes (comparisons between other groups are shown in Supplement File Figures S2–S10). It can be seen that PLA-DA identified two groups in pairs D–B (positive and negative ion modes), D–C (positive ion mode), E–A (positive ion mode), E–B (positive ion mode) and E–C (positive ion mode). However, OPLS-DAs completely identified all the groups. The VIP scores given by OPLS-DA were used to screen differential metabolites.

### 3.3. Screening for Differential Metabolites

The following steps constitute the screening process for differential metabolites: (1) a *t*-test statistical analysis was performed on the original data for every pair of time points, and the *ps* value was calculated; (2) the FC was calculated using the ratio of the relative quantitative mean for each pair of time points using the original data with thresholds FC > 2.0 or FC < 0.5; and (3) the VIP score was calculated based on the multivariate statistical analysis of the original data with a threshold value VIP > 1.0.

Differential metabolites were identified according to the preceding criteria. A total of 61 differential metabolites were identified in the positive ion mode and 57 differential metabolites were identified in the negative ion mode. A total of 117 differential metabolites were obtained after removing the repeated differential metabolites (Supplement File Table S3).

### 3.4. Bioinformatics Analysis

**Figure 4:**
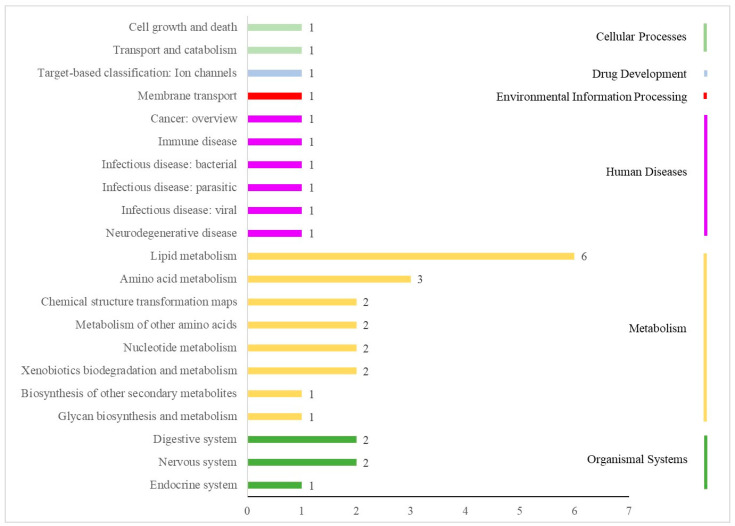
The KEGG analysis of differential metabolites. The abscissa is the number of differential metabolites involved.

**Figure 5:**
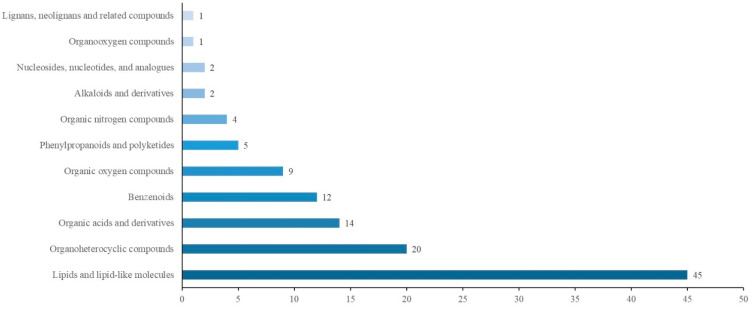
The HMDB annotations of differential metabolites. The abscissa is the number of differential metabolites involved.

**Figure 6:**
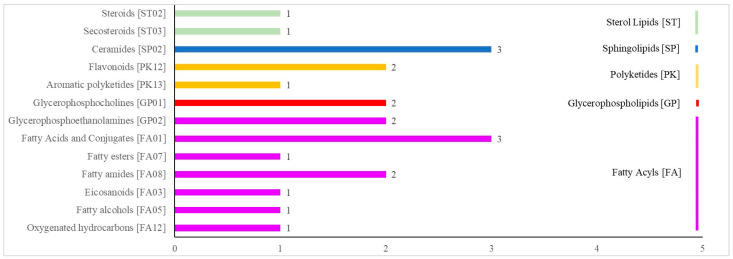
The LMSD annotations of differential metabolites. The abscissa is the number of differential metabolites involved.

Figure 4 shows the results of the KEGG analysis of 117 differential metabolites. There were 21 pathways in the following 6 categories: cellular processes, drug development, environmental information processing, human diseases, the metabolism and organismal systems. The HMDB annotations are shown in Figure 5 and included the following 11 categories: lipids and lipid-like molecules; organoheterocyclic compounds; organic acids and derivatives; benzenoids; organic oxygen compounds; phenylpropanoids and polyketides; organic nitrogen compounds; alkaloids and derivatives; nucleosides, nucleotides, and analogs; organooxygen compounds; and lignans, neolignans and related compounds. The LMSD annotations (Figure 6) included 5 categories (sterol lipids, sphingolipids, polyketides, glycerophospholipids and fatty acyls) and 13 comments (steroids, secosteroids, ceramides, flavonoids, aromatic polyketides, glycerophosphocholines, glycerophosphoethanolamines, fatty acids and conjugates, fatty esters, fatty amides, eicosanoids, fatty alcohols and oxygenated hydrocarbons). All differential metabolites identified are shown in Supplement File Tables S4–S6.

### 3.5. Identifying Key Differential Metabolites and Constructing the Metabolic Network

The bioinformatics analysis enabled us to identify 18 key differential metabolites related to reproduction, the lipid metabolism and the amino acid metabolism. There were nine differential metabolites related to an upregulated expression, six differential metabolites related to a downregulated expression and three differential metabolites related to an irregular expression over time. There were three differential metabolites related to reproduction, beta-cortol (Cor), tetrahydrocortisol (TH-Cor) and mycotoxin T2 (MT2); seven differential metabolites related to the lipid metabolism, phosphatidylserine (PS), triglyceride (TG), propanoic acid (Pac), 13S-hydroxyoctadecadienoic acid (13-HODE), 9S-hydroxyoctadecadienoic acid (9-HODE), phosphatidylcholine (PC) and phosphatidylethanolamine (PE); and eight differential metabolites related to the amino acid metabolism, arginyl-phenylalanine (Arg-Phe), D-glutamic acid (D-Glu), gamma-glutamylcysteine (Gln-Cys), glycylleucine (Gly), indolepyruvate (Ipa), 5-methoxytryptophol (Trp), tyrosyl-phenylalanine (Tyr-Phe) and tryptophyl-glutamine (Trp-Gln) (Table 1).

**Figure 7:**
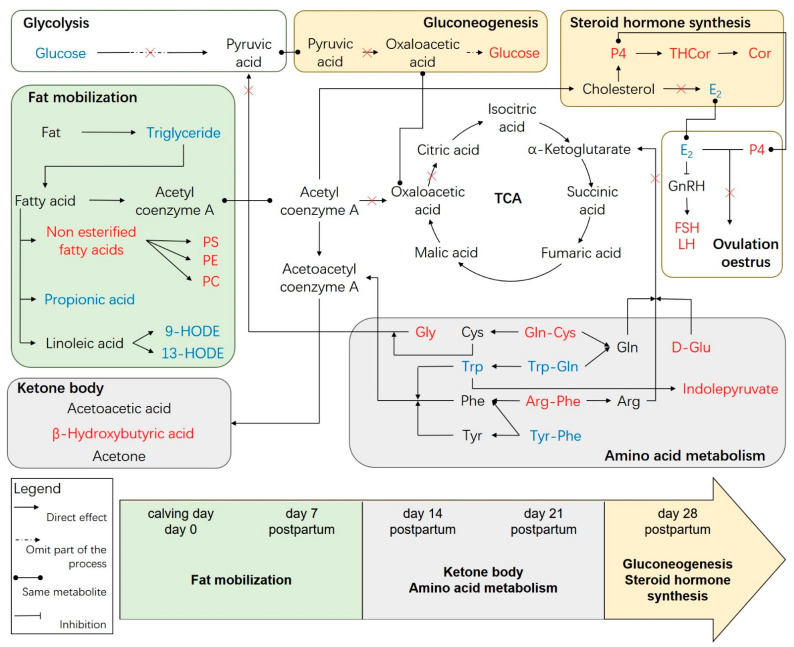
The metabolite network of female yaks during LPP. PS: a glycerol phospholipid metabolite of free fatty acids; PE: a glycerol phospholipid metabolite of free fatty acids; PC: a glycerol phospholipid metabolite of free fatty acids; 9-HODE: 9S-hydroxyoctadecadienoic acid;13-HODE: 13S-hydroxyoctadecadienoic acid; Gly: glycylleucine; Cys: cysteine; Gln: glutamine; D-Glu: D-glutamic acid; Trp: tryptophan; Phe: phenylalanine; Arg: arginine; Tyr: tryptophan; THCor: tetrahydrocortisol; Cor: beta-cortol; GnRH: gonadotropin-releasing hormone; E_2_: estradiol; P4: progesterone; FSH: follicle stimulating hormone; LH: luteinizing hormone; and TCA: tricarboxylic acid cycle. The metabolic process of the same background color indicates that it functions at the same time. The metabolites marked with red indicate that the expression is up regulated at the stage, while blue indicates that the expression is down regulated. “
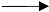
” direct effect; “
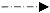
” omit part of the process; “
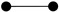
” same metabolite; and “
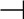
” inhibition; “×” bloked.

We diagrammed the LPP metabolite network of menstruating female yaks and the effects of the metabolites on the reproductive hormones based on the key differential metabolites we identified and their functions obtained by bioinformatics mining (Figure 7). The network includes glycolysis, the tricarboxylic acid cycle (TCA), gluconeogenesis, fat metabolism, ketone bodies, amino acid metabolism, steroid hormone production and ovulation estrus. We found that the main metabolic pathway in yaks was the fat metabolism from calving (day 0) until day 7 postpartum. The ketone body metabolism and ketogenic amino acids provided energy for the yaks from postpartum day 14 to postpartum day 21. Until postpartum day 28, the gluconeogenesis and steroid hormone synthesis pathways were active. However, glycolysis, TCA and ovulation estrus were not active due to the decreased glucose concentration.

## 4. Discussion

### 4.1. Glucose Metabolism in Yaks during LPP

Glucose is the primary body energy source of yaks [30]. The glucose metabolism consists primarily of glycolysis in anaerobic conditions and TCA in aerobic conditions. These two metabolic pathways decompose glucose to produce energy to supply the normal physiological needs of the body [31]. Glucose produces pyruvic acid through glycolysis. Synthesized pyruvic acid has two main pathways: one is to enter TCA to be decomposed to produce energy for the body; the other is through gluconeogenesis, which is the main mechanism of the glucose synthesis. It has long been confirmed that in domesticated cattle, the blood glucose content of females decreases due to the energy consumption by parturition on the day of birth [30]. When the blood glucose content decreases, the efficiency of glycolysis and TCA both decreases, and the body begins to synthesize glucose to provide energy [32]. However, as the efficiency of glycolysis decreases, the pyruvate production also decreases. The quantity of raw materials used in gluconeogenesis is insufficient to produce the quantities required by the body, and so the synthesis of glucose is blocked. It has been reported [33] that gluconeogenesis in domesticated cows is inhibited due to the reduced feed intake and increased lactation after delivery, which disrupts the blood glucose homeostasis [34]. As the glucose energy supply decreases, oxaloacetic acid accumulates in TCA. Oxaloacetic acid is a downstream metabolite of pyruvate in gluconeogenesis, so oxaloacetic acid is used in gluconeogenesis to synthesize glucose. This process leads to a trend in which glucose first decreases and then increases during the LPP of female yaks, which is consistent with the results we obtained in detecting reproductive hormones.

### 4.2. Lipid Metabolism of Female Yaks during LPP

Fat is the primary form of energy storage in yaks, principally as TG. TG decreased over time, which indicates that fatty acids were mobilized in female yaks during the LPP to provide energy to maintain the body’s needs [35]. Pac and linoleic acid are the same type of fatty acid, which is the first decomposition product. Pac directly affects adipocytes to regulate the metabolism of fatty acids [36,37]. 13-HODE and 9-HODE are derivatives of linoleic acid [38] and have been found in human studies related to the reproductive function [39]. However, there is no similar finding in yak research. We also found that several glycerophospholipids, which are produced through hydrolysis with non-esterified fatty acids, were expressed differently over time [40]. These results indicate that the quantities of fatty acids began to decrease at the end of the perinatal period. Meanwhile, when the glucose metabolism was in recovery, there was a consequent increase in the energy supply and the fat mobilization returned to normal. 

Fatty acids react to produce acetyl coenzyme A, which can be consumed in TCA for a complete oxidation, or it can be used to synthesize fatty acids through the reverse pathway of the fatty acid metabolism [41]. However, acetyl coenzyme A does not participate in either of these processes in female yaks during the LPP due to the stagnation of the glucose metabolism and the intensity of the fat mobilization. In order to provide energy, acetyl coenzyme A is converted into acetoacetyl coenzyme A, which then forms the ketone body (acetoacetic acid, β-hydroxybutyric acid and acetone) [42]. Ketone bodies compensate for the negative energy balance in female cows during the LPP. This shows that yaks also have a negative energy balance during the LPP, which is revealed for the first time in this study.

We identified that the female yak energy supply was insufficient to meet the body’s energy requirements in the early stage of the perinatal period and there was a large negative energy balance. The fat metabolism was active to supplement energy, but an excessive fatty acid mobilization produces acetyl coenzyme A that in turn produces ketone bodies.

### 4.3. Amino Acid Metabolism of Female Yaks during LPP

The protein metabolism also provides energy to reduce the energy gap in the body [43]. Amino acids are the basic units of proteins and are categorized as glucogenic amino acids or ketogenic amino acids.

#### 4.3.1. Glucogenic Amino Acids

Glucogenic amino acids are converted to pyruvate or α-ketoglutarate and enter gluconeogenesis and TCA, respectively. Cys is converted to pyruvate and provides a carbon source for the TCA [44]; Gln and D-Glu are converted to α-ketoglutarate, which is formed by a transamination after the oxidation of α-ketoglutarate and enters TCA [45,46]. Arg and Gly is converted into the intermediate product of gluconeogenesis through the deamination to produce glucose.

The glucogenic amino acids and amino acid complexes identified in the experiment were upregulated, which indicates that the efficiency of the amino acid metabolism increased to supplement the blood glucose when the blood glucose was low. However, due to the low efficiency of gluconeogenesis and TCA at the beginning of the perinatal period, glucogenic amino acids were inactive and accumulated, thus increasing in serum over the period.

#### 4.3.2. Ketogenic Amino Acids

We also found ketogenic amino acids including Trp, Tyr and Phe. Ketogenic amino acids promote acetoacetyl coenzyme A to produce ketone bodies. Trp is converted to pyruvate, which decarboxylates to produce acetoacetic acid [47]; Tyr synthesizes fumaric acid and acetoacetic acid through an oxidation and hydrolysis [48,49]. Phe produces acetoacetic acid through tyrosine aminotransferase catalysis [50,51].

We found that ketogenic amino acids and amino acid complexes were both upregulated and downregulated. We infer that the reason for these opposing expressions of Phe and other ketogenic amino acids was as follows. In the early postpartum period, the Arg complex was produced in large quantities through glucogenic paths but was not accumulated due to gluconeogenesis. The downregulation of Tyr and Trp indicates that Tyr and Trp participate in the initiation of the mobilization of fatty acids, and the acetyl coenzyme A produced was largely converted to ketones to provide energy for the body.

### 4.4. Synthesis and Secretion of Reproductive Hormones in Female Yaks during LPP

The secretion and synthesis of reproductive hormones during the LPP indicates the recovery of the female yak reproductive system and to some extent the time of the next pregnancy. A loss of energy during the LPP, the impaired glucose metabolism and an excessive fat mobilization resulted in the body producing ketones to supply energy, resulting in a negative energy balance. Acetyl coenzyme A, that was produced by an excessive fatty acid mobilization, is also a constituent of cholesterol in the blood’s circulation. Steroid hormones are cyclic aliphatic hydrocarbons synthesized by cholesterol through enzymatic hydrolysis reactions and consist mainly of glucocorticoids, mineralocorticoids and gonadal corticoids [52]. Cholesterol in circulating blood primarily acts on gonads and is critical to the production of gametes and the secretion of gonadal hormones [53]. Studies have shown that cholesterol can be metabolized after catalysis to synthesize steroid hormones, cortisol and progesterone [52]. A mouse study showed that after a severe diet restriction, the metabolic pathway of cholesterol to steroid hormones in adrenal cells was active and a large quantity of cortisol was synthesized to adapt to various external stresses [54]. These cited results are consistent with the results of this study. The metabolomic results show that TH-cor and Cor, the terminal products of P4 synthesis, significantly increased at times D and E, indicating that P4 had been formed and began to produce terminal products during the later stage of the LPP. Cholesterol can also generate E_2_, the most important estrogen. The synthesis of the ovarian steroid hormone E_2_ is regulated by a variety of proteins [55]. After acting on the central nervous system, E_2_ cooperates with P4 to promote uterine development and induce estrus in female mammals. MT2 was also found among the various metabolites. Several research reports found that milk production and the estrus of dairy cattle are related to MT2 [56]. However, the relevant mechanism is not clear.

We hypothesize that, based on the research, the small quantity of acetyl coenzyme A that was produced after the excessive fat mobilization formed cholesterol in addition to the large quantity of ketones, while only cholesterol formed P4, leading to the upregulation of the final products TH-cor and Cor. However, cholesterol does not synthesize a large quantity of E_2_, which is consistent with the detection of reproductive hormones. The concentrations of the reproductive hormones that regulate estrus were not equal during the LPP, which may be the main reason why the recovery of the reproductive system in female yaks after parturition is not rapid and thus there is a long calving interval. We hope that these results will enable yak breeders to pay more attention to feeding during the LPP. In the LPP, a supplement of GLU, cholesterol or E_2_ may accelerate the recovery of the reproductive system of yaks after delivery. However, the specific mechanism needs to be further verified.

## 5. Conclusions

In this study, we analyzed the immediate postpartum blood metabolism profiles of female yaks. Through the identification of key metabolites and bioinformatics analysis, we found that the postpartum female yaks had a negative energy balance, and that the glucose metabolism, lipid metabolism and amino acid metabolism were all disordered. The disorders influenced the synthesis and secretion of reproductive hormones, which impeded the postpartum recovery of the reproductive system. The specific mechanisms we hypothesized need a further verification. This study provides basic information and data for the nutrient metabolism and the reproductive capability of female yaks in plateau areas and indicates a new direction for research into the farming and management of yaks.

## Figures and Tables

**Table 1:** The key differential metabolites.

HMDB_ID	Description	Abbreviation	UP or DOWN	
HMDB0005821	Beta-cortol	Cor	E/A	UP
HMDB0000949	Tetrahydrocortisol	TH-Cor	D/B	UP
HMDB0032864	Mycotoxin T 2	MT2	E/A	DOWN
HMDB0010166	PS(18:0/22:5(7Z,10Z,13Z,16Z,19Z))	PS	C/A E/A	UP UP
HMDB0043117	TG(15:0/22:0/22:1(13Z))	TG	C/A	UP
HMDB0008850	PE(14:0/P-16:0)	PE	C/A	UP
HMDB0030254	Propanoic acid	Pac	E/A E/C E/D	DOWN DOWN DOWN
HMDB0004667	13S-hydroxyoctadecadienoic acid	13-HODE	D/A	DOWN
HMDB0006940	9(S)-HPODE	9-HODE	D/A D/B	DOWN DOWN
HMDB0007931	PC(14:1(9Z)/P-18:1(9Z))	PC	E/C	DOWN
HMDB0007961	PC(15:0/P-16:0)	PC	C/A E/B E/C	UP DOWN DOWN
HMDB0008064	PC(18:1(11Z)/14:0)	PC	D/C E/A E/B E/C	DOWN DOWN DOWN DOWN
HMDB0008394	PC(20:3(8Z,11Z,14Z)/14:0)	PC	D/C E/C	DOWN DOWN
HMDB0008892	PE(15:0/18:0)	PE	E/A E/C E/D	DOWN DOWN DOWN
HMDB0008916	PE(15:0/P-16:0)	PE	E/C	DOWN
HMDB0009048	PE(18:1(11Z)/P-16:0)	PE	D/C E/C	DOWN DOWN
HMDB0009378	PE(20:3(8Z,11Z,14Z)/P-16:0)	PE	E/C	DOWN
HMDB0011386	PE(P-18:0/20:4(8Z,11Z,14Z,17Z))	PE	E/C	DOWN
HMDB0011401	PE(P-18:1(11Z)/14:0)	PE	E/C	DOWN
HMDB0011403	PE(P-18:1(11Z)/15:0)	PE	E/C	DOWN
HMDB0011416	PE(P-18:1(11Z)/20:3(5Z,8Z,11Z))	PE	E/C	DOWN
HMDB0010569	PE-NMe(16:0/18:1(9Z))	PE	E/B E/C	DOWN DOWN
HMDB0028716	Arginyl-phenylalanine	Arg-Phe	C/A	UP
HMDB0003339	D-glutamic acid	D-Glu	D/B	UP
HMDB0001049	Gamma-glutamylcysteine	Gln-Cys	E/B	UP
HMDB0000759	Glycylleucine	Gly	D/A E/A	UP UP
HMDB0060484	Indolepyruvate	Ipa	E/A	UP
HMDB0001896	5-Methoxytryptophol	Trp	E/C	DOWN
HMDB0029081	Tryptophyl-glutamine	Trp-Gln	C/A D/A E/D	DOWN DOWN UP

Note: A. B, C, D and E represent the grouping of 5 time points; DOWN indicates that the expression of metabolites is down regulated in the group represented by molecules compared with the group represented by denominator. Similarly, UP expression was up regulated. For example, “E/A, DOWN” indicates that the expression of this metabolite is down regulated in group E compared with group A.

## Data Availability

The data presented in this study are available in articles and Supplementary Material.

## Supplementary Materials

The following supporting information can be downloaded at: https://www.mdpi.com/article/10.3390/metabo12111113/s1. Figure S1: The correlation analysis of the QC samples in positive and negative ion modes. A is positive ion modes; B is negative ion modes.; Figure S2: PLS-DA, OPLS-DA, VIP scores and cluster diagram analysis results taking B/A. The figures in the first line (A–D) are the analysis results in the positive ion mode, which are, respectively, PLS-DA, OPLS-DA, VIP scores and cluster diagram analysis; the second line (E–H) are the analysis results in the negative ion mode; Figure S3: PLS-DA, OPLS-DA, VIP scores and cluster diagram analysis results taking C/A. The figures in the first line (A–D) are the analysis results in the positive ion mode, which are, respectively, PLS-DA, OPLS-DA, VIP scores and cluster diagram analysis; the second line (E–H) are the analysis results in the negative ion mode; Figure S4: PLS-DA, OPLS-DA, VIP scores and cluster diagram analysis results taking C/B. The figures in the first line (A–D) are the analysis results in the positive ion mode, which are, respectively, PLS-DA, OPLS-DA, VIP scores and cluster diagram analysis; the second line (E–H) are the analysis results in the negative ion mode; Figure S5: PLS-DA, OPLS-DA, VIP scores and cluster diagram analysis results taking D/A. The figures in the first line (A–D) are the analysis results in the positive ion mode, which are, respectively, PLS-DA, OPLS-DA, VIP scores and cluster diagram analysis; the second line (E–H) are the analysis results in the negative ion mode; Figure S6: PLS-DA, OPLS-DA, VIP scores and cluster diagram analysis results taking D/B. The figures in the first line (A–D) are the analysis results in the positive ion mode, which are, respectively, PLS-DA, OPLS-DA, VIP scores and cluster diagram analysis; the second line (E–H) are the analysis results in the negative ion mode; Figure S7: PLS-DA, OPLS-DA, VIP scores and cluster diagram analysis results taking D/C. The figures in the first line (A–D) are the analysis results in the positive ion mode, which are, respectively, PLS-DA, OPLS-DA, VIP scores and cluster diagram analysis; the second line (E–H) are the analysis results in the negative ion mode; Figure S8: PLS-DA, OPLS-DA, VIP scores and cluster diagram analysis results taking E/A. The figures in the first line (A–D) are the analysis results in the positive ion mode, which are, respectively, PLS-DA, OPLS-DA, VIP scores and cluster diagram analysis; the second line (E–H) are the analysis results in the negative ion mode; Figure S9: PLS-DA, OPLS-DA, VIP scores and cluster diagram analysis results taking E/B. The figures in the first line (A–D) are the analysis results in the positive ion mode, which are, respectively, PLS-DA, OPLS-DA, VIP scores and cluster diagram analysis; the second line (E–H) are the analysis results in the negative ion mode; Figure S10: PLS-DA, OPLS-DA, VIP scores and cluster diagram analysis results taking E/D. The figures in the first line (A–D) are the analysis results in the positive ion mode, which are, respectively, PLS-DA, OPLS-DA, VIP scores and cluster diagram analysis; the second line (E–H) are the analysis results in the negative ion mode; Table S1: The information of experimental animals; Table S2: The detection results of major serum compounds during LPP; Table S3: Qualitative results of differential metabolites; Table S4: The result of KEGG analysis; Table S5:The result of HMDB analysis; Table S6:The result of LMSD analysis.
