# Supplementary material for: The Effects of Postpartum Yak Metabolism on Reproductive System Recovery
Source: Metabolites. 2022 Nov 15;12(11):1113. doi: 10.3390/metabo12111113 (PMC9694671; doi:10.3390/metabo12111113)
Supplement: Supplementary file 1 [file metabolites-12-01113-s001.zip › metabolites-2009471-supplementary.pdf]

Supplementary Materials

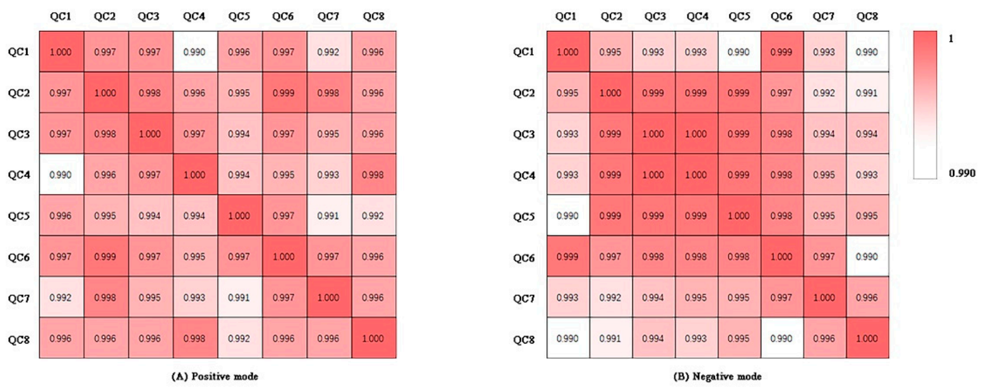

Figure S1. The correlation analysis of the QC samples in positive and negative ion modes. A is positive ion modes; B is negative ion modes.

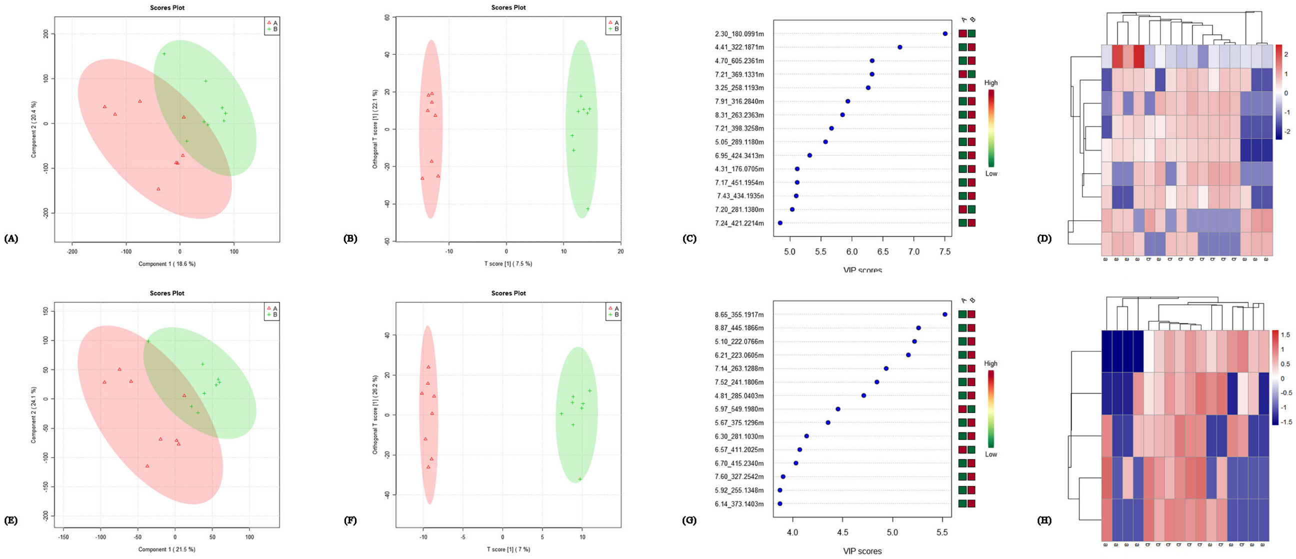

Figure S2. PLS-DA, OPLS-DA, VIP scores and cluster diagram analysis results taking B/A; The figures in the first line (A–D) are the analysis results in the positive ion mode, which are, respectively, PLS-DA, OPLS-DA, VIP scores and cluster diagram analysis; the second line (E–H) are the analysis results in the negative ion mode.

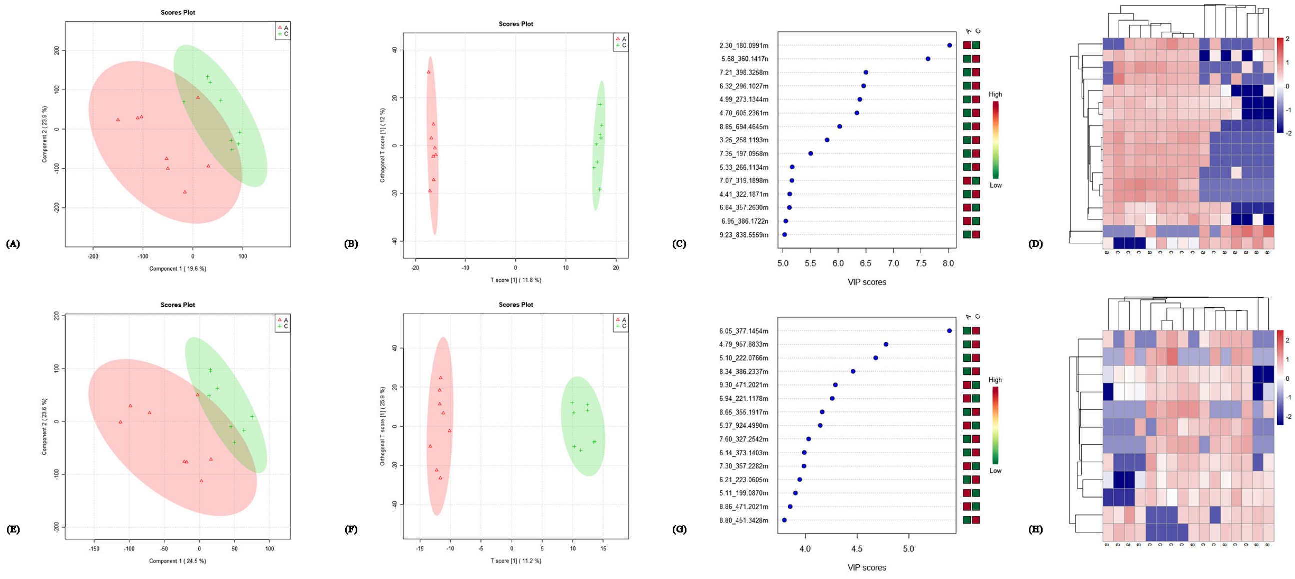

Figure S3. PLS-DA, OPLS-DA, VIP scores and cluster diagram analysis results taking C/A; The figures in the first line (A–D) are the analysis results in the positive ion mode, which are, respectively, PLS-DA, OPLS-DA, VIP scores and cluster diagram analysis; the second line (E–H) are the analysis results in the negative ion mode.

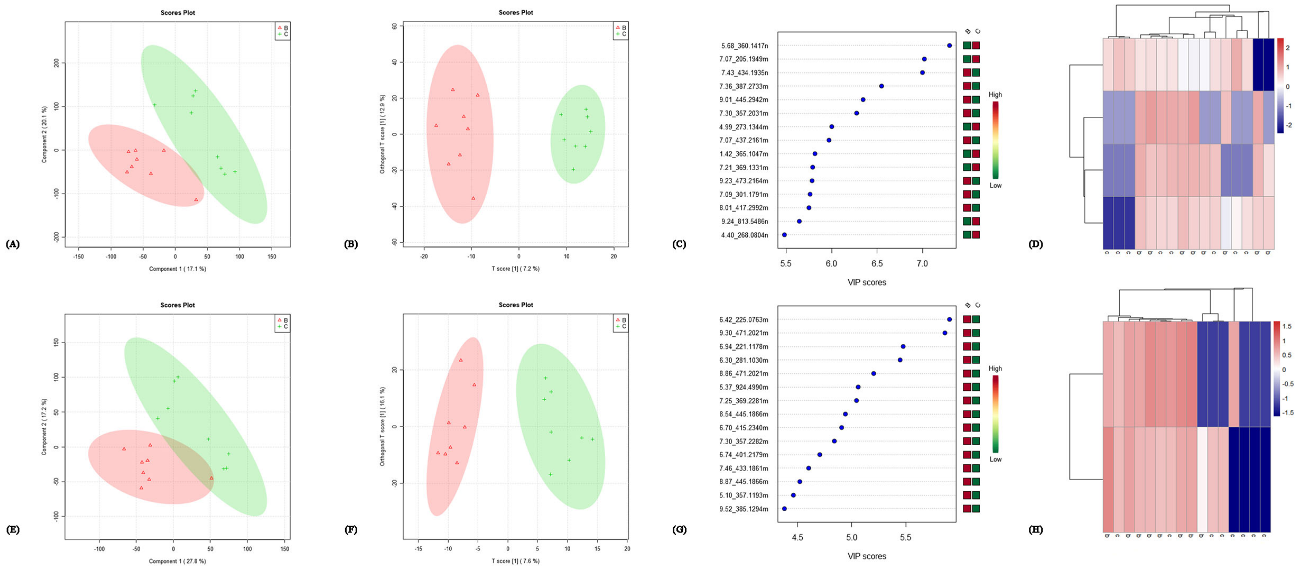

Figure S4. PLS-DA, OPLS-DA, VIP scores and cluster diagram analysis results taking C/B; The figures in the first line (A–D) are the analysis results in the positive ion mode, which are, respectively, PLS-DA, OPLS-DA, VIP scores and cluster diagram analysis; the second line (E–H) are the analysis results in the negative ion mode.

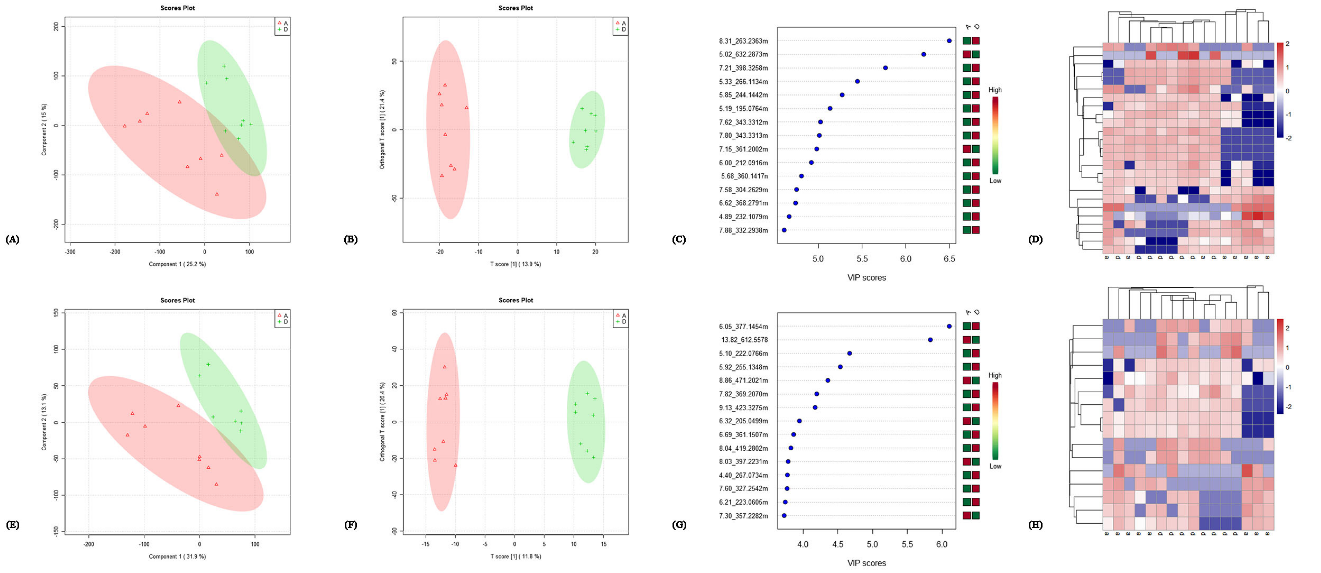

Figure S5. PLS-DA, OPLS-DA, VIP scores and cluster diagram analysis results taking D/A; The figures in the first line (A–D) are the analysis results in the positive ion mode, which are, respectively, PLS-DA, OPLS-DA, VIP scores and cluster diagram analysis; the second line (E–H) are the analysis results in the negative ion mode.

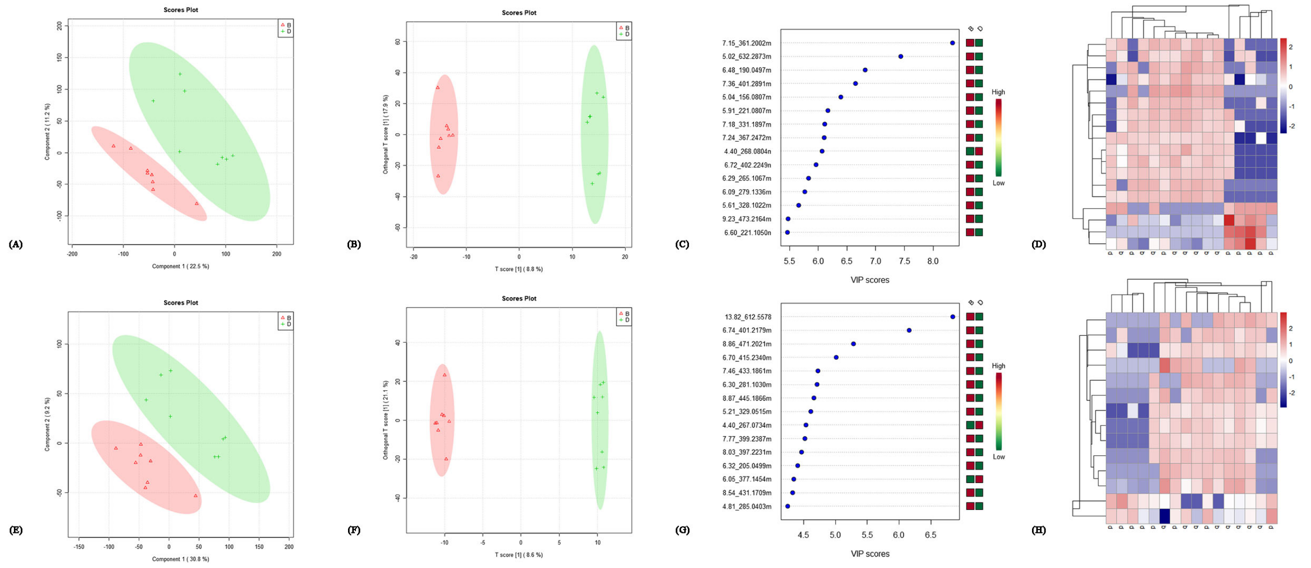

Figure S6. PLS-DA, OPLS-DA, VIP scores and cluster diagram analysis results taking D/B; The figures in the first line (A–D) are the analysis results in the positive ion mode, which are, respectively, PLS-DA, OPLS-DA, VIP scores and cluster diagram analysis; the second line (E–H) are the analysis results in the negative ion mode.

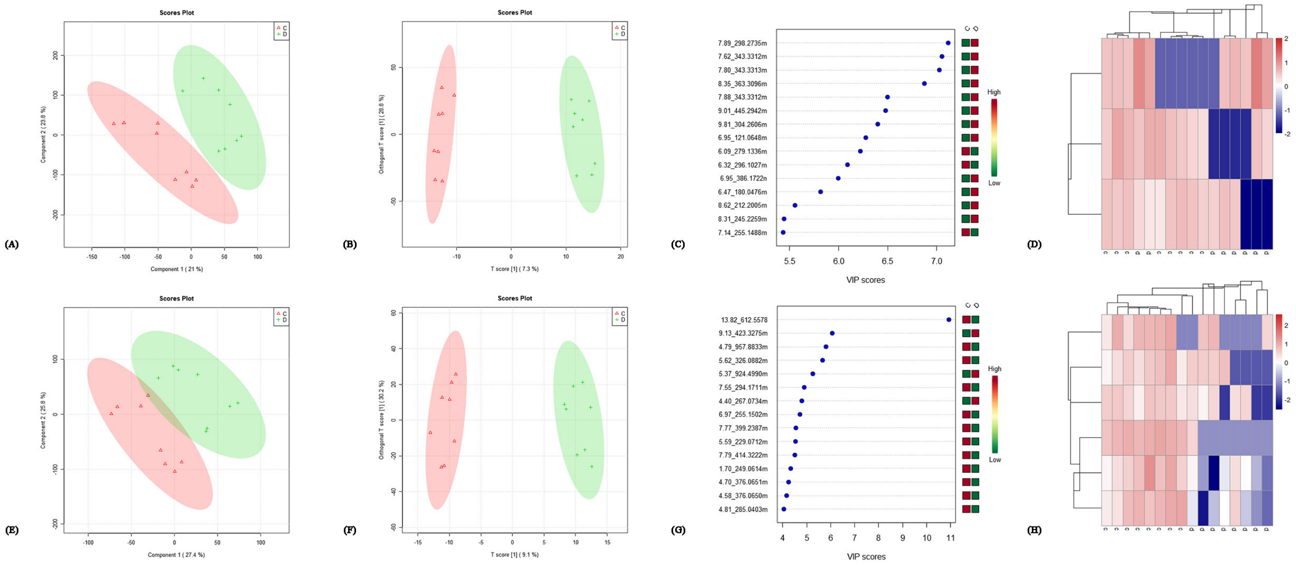

Figure S7. PLS-DA, OPLS-DA, VIP scores and cluster diagram analysis results taking D/C; The figures in the first line (A–D) are the analysis results in the positive ion mode, which are, respectively, PLS-DA, OPLS-DA, VIP scores and cluster diagram analysis; the second line (E–H) are the analysis results in the negative ion mode.

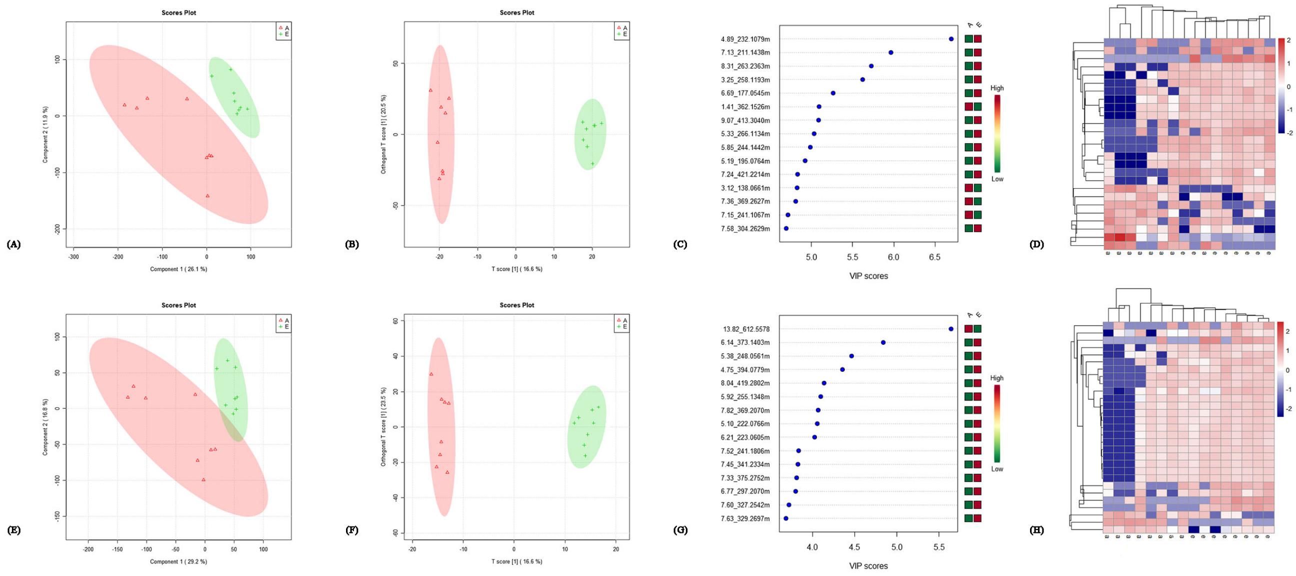

Figure S8. PLS-DA, OPLS-DA, VIP scores and cluster diagram analysis results taking E/A; The figures in the first line (A–D) are the analysis results in the positive ion mode, which are, respectively, PLS-DA, OPLS-DA, VIP scores and cluster diagram analysis; the second line (E–H) are the analysis results in the negative ion mode.

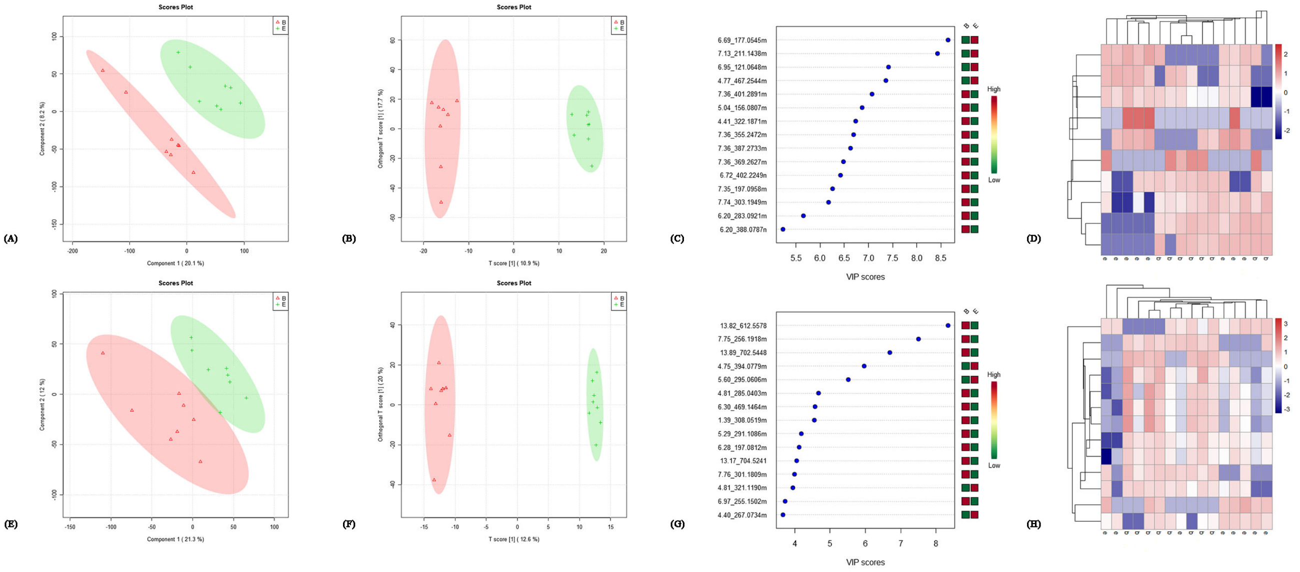

Figure S9. PLS-DA, OPLS-DA, VIP scores and cluster diagram analysis results taking E/B; The figures in the first line (A–D) are the analysis results in the positive ion mode, which are, respectively, PLS-DA, OPLS-DA, VIP scores and cluster diagram analysis; the second line (E–H) are the analysis results in the negative ion mode.

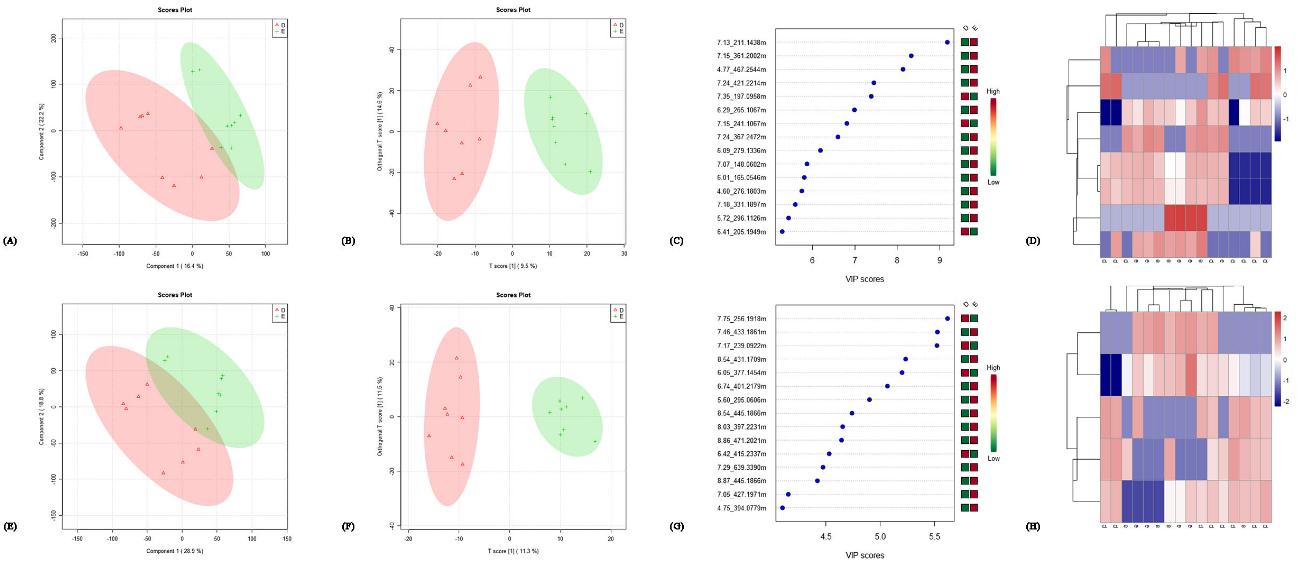

Figure S10. PLS-DA, OPLS-DA, VIP scores and cluster diagram analysis results taking E/D; The figures in the first line (A–D) are the analysis results in the positive ion mode, which are, respectively, PLS-DA, OPLS-DA, VIP scores and cluster diagram analysis; the second line (E–H) are the analysis results in the negative ion mode.

Table S1. The information of experimental animals.

No.	Age	Parity	Body condition (1-5)	Applied to LC-MS
50942	7	1	3	Applied
50618	8	2	3	No-Applied
40546	8	2	3	Applied
50606	6	1	2	No-Applied
40548	8	2	2	No-Applied
50632	7	2	2	Applied
50622	6	2	3	No-Applied
50609	6	2	3	No-Applied
45636	5	1	2	Applied
47894	6	1	2	No-Applied
50287	7	1	2	No-Applied
48995	6	1	2	No-Applied
56055	6	1	3	No-Applied
52990	6	1	3	Applied
57058	7	1	2	Applied
48095	6	1	3	No-Applied
58438	6	1	3	No-Applied
47637	5	1	2	No-Applied
55915	8	2	2	Applied
51461	5	1	3	No-Applied
42758	8	2	2	No-Applied
58067	5	1	3	Applied
40567	6	1	2	No-Applied
54080	6	1	3	No-Applied
52864	5	1	3	No-Applied
Mean	6.36	1.32	2.52	-
SD	1.04	0.48	0.51	-

Table S2. The detection results of major serum compounds during LPP.

		GLU (mmol/L)			
**No.**	**A**	**B**	**C**	**D**	E
50942	3.15	3.34	3.02	2.96	2.67
50618	2.77	3.04	3.02	3.81	3.54
40546	2.71	3.4	3.57	2.98	3.54
50606	3.59	2.73	2.45	1.74	4.17
40548	3.38	3.34	2.14	2.98	2.69
50632	2.96	2.35	3.11	2.69	5.77
50622	3	3.36	3.53	2.38	3.02
50609	2.49	3.36	2.58	3.46	3.11
45636	2.75	2.33	3.04	3.75	4.17
47894	3.38	3.3	3.66	2.98	3.38
50287	3.15	4.04	3.32	4.19	2.61
48995	2.81	3.49	3.64	3.88	3.15
56055	3.28	3.02	3.74	3.84	3.44
52990	3.76	3	2.79	2.15	3.34
57058	2.54	3.17	2.9	2.32	3.52
48095	3.38	3.78	2.98	3.4	2.57
58438	3.68	3.74	2.79	2.84	3.32
47637	3.59	3.89	2.77	2.59	3.21
55915	3.51	2.07	3.7	3.63	2.92
51461	3.3	2.54	3.72	4.04	3.46
42758	2.73	2.5	3.7	3.69	4.79
58067	2.64	3.5	2.6	4.6	5.79
40567	4.08	3.17	3.17	3.13	3.46
54080	2.45	2.63	4.79	4.35	3.63
52864	3.28	3.11	2.19	4.54	3.09
Mean	3.134	3.128	3.156	3.316	3.534
SD	0.440	0.513	0.592	0.767	0.844
Sig.	A	A	A	A	B
Mean±SD	3.134±0.44	3.128±0.513	3.156±0.592	3.316±0.767	3.534±0.844

		TG (mmol/L)			
No.	A	B	C	D	E
50942	0.92	0.74	0.57	0.43	0.44
50618	0.87	0.78	0.5	0.41	0.56
40546	0.83	0.8	0.48	0.63	0.55
50606	0.8	0.83	0.51	0.56	0.5
40548	0.83	0.62	0.47	0.43	0.39
50632	0.92	0.91	0.5	0.45	0.45
50622	0.85	0.65	0.53	0.58	0.45
50609	0.79	0.73	0.5	0.38	0.53
45636	1.16	0.52	0.53	0.5	0.46
47894	1.02	0.65	0.63	0.51	0.47
50287	0.89	0.68	0.43	0.51	0.48
48995	0.8	0.65	0.5	0.55	0.42
56055	0.73	0.59	0.42	0.54	0.4
52990	0.77	0.81	0.62	0.48	0.42
57058	0.67	0.76	0.52	0.36	0.39
48095	0.69	0.49	0.49	0.65	0.42
58438	0.88	0.53	0.48	0.43	0.44
47637	0.7	0.54	0.55	0.48	0.41

55915	0.79	0.63	0.55	0.35	0.44
51461	0.77	0.66	0.49	0.34	0.5
42758	0.74	0.55	0.58	0.44	0.44
58067	0.67	0.56	0.57	0.42	0.39
40567	0.69	0.66	0.53	0.4	0.46
54080	0.67	0.73	0.52	0.46	0.39
52864	0.7	0.73	0.57	0.39	0.43
Mean	0.806	0.672	0.521	0.467	0.449
SD	0.118	0.109	0.051	0.083	0.048
Sig.	A	B	C	D	D
Mean±SD	0.806±0.118	0.672±0.109	0.521±0.051	0.467±0.083	0.449±0.048
NEFA (mmol/L)					
No.	A	B	C	D	E
50942	0.52	2.86	1.76	0.58	0.45
50618	0.44	1.84	2.72	0.51	0.53
40546	0.53	1.6	2.33	0.26	0.3
50606	0.4	2.11	1.98	0.21	0.53
40548	0.54	1.46	2.12	0.32	0.3
50632	0.49	1.64	1.6	0.38	0.47
50622	0.47	1.23	1.75	0.6	0.64
50609	0.5	2.11	1.81	0.67	0.38
45636	0.4	2.03	1.25	0.58	0.27
47894	0.51	2.65	1.31	0.55	0.42
50287	0.67	2.19	1.75	0.42	0.27
48995	0.44	1.19	1.64	0.38	0.32
56055	0.49	0.94	2.18	0.37	0.61
52990	0.42	1.16	2.26	0.6	0.39
57058	0.37	2.36	2.27	0.2	0.31
48095	0.41	2.39	1.36	0.23	0.27
58438	0.46	2.2	1.41	0.44	0.25
47637	0.46	2.08	1.96	0.38	0.22
55915	0.49	1.85	1.72	0.31	0.45
51461	0.46	2.11	2.35	0.13	0.25
42758	0.41	1.7	1.37	0.57	0.46
58067	0.43	2.92	2.75	0.37	0.2
40567	0.49	1.93	1.58	0.12	0.35
54080	0.4	2.31	1.68	0.36	0.28
52864	0.44	1.24	1.8	0.18	0.27
Mean	0.465	1.924	1.868	0.388	0.367
SD	0.062	0.529	0.416	0.161	0.122
Sig.	A	B	B	A	A
Mean±SD	0.465±0.062	1.924±0.529	1.868±0.416	0.388±0.161	0.367±0.122
BHBA (mmol/L)					
No.	A	B	C	D	E
50942	1.57	2.86	2.33	0.02	0.02
50618	1.63	1.84	1.98	0.03	0.02
40546	1.31	1.60	2.12	0.03	0.01
50606	1.98	2.11	1.6	0.02	0.02
40548	1.47	1.46	1.75	0.02	0.02
50632	1.28	1.64	1.81	0.01	0.02
50622	1.54	1.23	1.25	0.02	0.02
50609	1.60	2.11	1.31	0.02	0.01

45636	1.82	2.03	1.75	0.02	0.02
47894	1.46	2.65	1.64	0.02	0.02
50287	1.54	2.19	2.18	0.02	0.02
48995	1.68	1.19	2.26	0.03	0.01
56055	1.32	0.94	2.27	0.02	0.03
52990	1.44	1.16	1.36	0.02	0.02
57058	1.79	2.36	1.41	0.01	0.02
48095	2.22	2.39	1.96	0.02	0.02
58438	1.84	2.20	3.3	0.02	0.02
47637	1.79	2.08	1.72	0.01	0.02
55915	1.77	1.85	2.35	0.02	0.02
51461	1.71	2.11	1.15	0.01	0.02
42758	1.62	1.70	1.37	0.01	0.01
58067	2.33	2.92	2.75	0.02	0.02
40567	1.53	1.93	2.14	0.02	0.01
54080	1.08	1.76	2.31	0.02	0.02
52864	1.57	2.72	1.24	0.01	0.02
Mean	1.635	1.961	1.892	0.018	0.018
SD	0.279	0.530	0.521	0.005	0.004
Sig.	A	B	B	C	C
Mean±SD	1.635±0.279	1.961±0.53	1.892±0.521	0.018±0.005	0.018±0.004

TP(g/L)					
No.	A	B	C	D	E
50942	1.87	1.51	2.89	1.97	2.08
50618	2.15	2.77	3.09	2.79	2.27
40546	0.92	2.13	2.25	3.02	2.33
50606	1.83	2.79	2.83	2.56	2.16
40548	1.55	2.51	2.45	1.66	2.08
50632	1	3.05	3.51	2	3.08
50622	1.65	3.21	2.53	1.77	0.62
50609	1.75	2.09	4.24	1.54	2.41
45636	0.8	2.81	3.47	1.14	2.04
47894	2.25	1.99	2.71	0.97	2.06
50287	2.49	2.49	2.65	1.45	2.56
48995	2.07	2.81	2.77	0.95	1.49
56055	1.37	3.11	3.27	0.99	2.79
52990	1.16	3.07	3.35	1.56	1.47
57058	1.24	2.99	4.74	1.41	2.77
48095	0.96	2.73	4.72	0.85	1.35
58438	2.81	2.29	3.33	2.12	2.02
47637	1.39	2.31	3.96	1.6	1.35
55915	0.98	2.04	2.87	2.12	1.83
51461	1.18	2.06	3.61	2.85	1.41
42758	1.75	2.37	2.87	1.66	1.12
58067	1.75	2.09	3.65	1.91	0.82
40567	1.79	2.25	3.09	1.18	1.54
54080	2.01	2	3.51	1.14	2.06
52864	1.93	2.41	3.03	0.82	2.16
Mean	1.626	2.475	3.255	1.681	1.914
SD	0.522	0.440	0.648	0.637	0.611
Sig.	A	B	C	A	A
Mean±SD	1.626±0.522	2.475±0.44	3.255±0.648	1.681±0.637	1.914±0.611

E$_2$(nmol/L)					
No.	A	B	C	D	E
50942	0.27	0.21	0.17	0.22	0.17
50618	0.21	0.38	0.5	0.16	0.16
40546	0.25	0.13	0.42	0.19	0.22
50606	0.35	0.28	0.29	0.21	0.22
40548	0.44	0.2	0.27	0.22	0.23
50632	0.07	0.35	0.12	0.24	0.25
50622	0.19	0.46	0.19	0.18	0.14
50609	0.26	0.36	0.19	0.19	0.15
45636	0.37	0.34	0.2	0.19	0.15
47894	0.4	0.45	0.41	0.16	0.15
50287	0.38	0.42	0.27	0.12	0.15
48995	0.18	0.42	0.59	0.11	0.16
56055	0.05	0.36	0.35	0.17	0.11
52990	0.14	0.31	0.46	0.15	0.17
57058	0.16	0.32	0.49	0.11	0.23
48095	0.17	0.3	0.65	0.16	0.19
58438	0.33	0.33	0.27	0.16	0.14
47637	0.15	0.25	0.14	0.2	0.16
55915	0.07	0.23	0.5	0.31	0.15
51461	0.18	0.24	0.41	0.26	0.15
42758	0.27	0.32	0.42	0.17	0.12
58067	0.31	0.37	0.16	0.19	0.12
40567	0.36	0.35	0.17	0.14	0.16
54080	0.13	0.29	0.28	0.16	0.17
52864	0.23	0.35	0.23	0.15	0.2
Mean	0.236	0.320	0.326	0.180	0.168
SD	0.109	0.080	0.150	0.045	0.037
P	A	B	B	C	C
Mean±SD	0.236±0.109	0.32±0.08	0.326±0.15	0.18±0.045	0.168±0.037

P$_4$(nmol/L)					
No.	A	B	C	D	E
50942	2.08	0.75	1.16	1.42	1.24
50618	2.13	3.23	1.44	3.25	1.58
40546	2.99	1.01	1.03	1.92	2.11
50606	3.05	0.88	0.73	1.85	1.49
40548	2.6	1.63	0.73	1.19	1.8
50632	1.16	0.79	3.1	1.5	1.81
50622	1.91	3.36	2.13	1.15	1.5
50609	1.83	1.09	1.63	1.51	1.89
45636	1.46	1.7	1.93	2.39	1.53
47894	1.76	1.7	1.33	1.62	1.84
50287	2.06	1.67	0.51	2.35	1.68
48995	3.59	2.06	0.49	2.55	1.68
56055	1.72	1.65	1.07	2.4	1.5
52990	2.82	1.2	1.42	1.67	1.58
57058	1.57	2.95	1.33	1.45	2.76
48095	2.58	1.44	2.08	2.4	1.74
58438	1.57	1.65	2.11	1.55	3.33
47637	0.86	1.07	1.42	1.49	1.73
55915	0.77	2.23	2.32	1.69	1.82

51461	0.49	2.58	1.01	1.31	1.11
42758	0.96	0.98	0.45	1.81	1.61
58067	2.34	1.95	2.45	3.05	1.31
40567	1.7	1.95	2.32	1.79	1.72
54080	1.05	2.13	0.47	1.6	1.61
52864	0.7	2.84	1.44	1	1.15
Mean	1.830	1.779	1.444	1.836	1.724
SD	0.811	0.758	0.713	0.575	0.467
P	A	AB	B	A	AB
Mean±SD	1.83±0.811	1.779±0.758	1.444±0.713	1.836±0.575	1.724±0.467
			FSH (ug/L)		
No.	A	B	C	D	E
50942	5.42	0.83	3.03	5.58	5.19
50618	3.95	9.93	1.19	7.44	6.71
40546	5.7	5.7	5.42	7.64	4.8
50606	2.67	5.15	4.23	6.29	5.53
40548	2.3	6.25	1.19	5.09	5.68
50632	9.38	3.86	7.17	5.48	9.85
50622	7.08	1.1	3.03	7.05	5.65
50609	3.77	0.74	2.76	7.3	6.24
45636	1.1	3.31	2.94	7	6.02
47894	5.06	0.09	4.32	6.46	7.22
50287	2.3	8	3.31	4.99	5.07
48995	1.29	0.28	5.33	9.4	6.27
56055	2.67	2.48	6.53	5.88	6.49
52990	2.94	1.56	1.75	7.03	5.58
57058	3.03	3.4	2.21	5.73	7.2
48095	4.04	2.57	2.3	6.05	6.86
58438	7.26	4.5	5.97	5.53	8.25
47637	6.62	5.43	6.62	4.92	6.83
55915	6.62	6.39	3.68	6.63	6.14
51461	7.26	4.36	4.6	7.44	5.16
42758	5.43	8.23	7.72	4.85	6.95
58067	5.29	6.34	4.04	7.96	6.29
40567	5.53	5.97	5.02	6.1	5.78
54080	5.16	5.12	4.38	6.66	5.09
52864	5.21	5.61	5.48	5.53	7.27
Mean	4.683	4.288	4.168	6.401	6.324
SD	2.079	2.637	1.826	1.109	1.122
P	A	A	A	B	B
Mean±SD	4.683±2.079	4.288±2.637	4.168±1.826	6.401±1.109	6.324±1.122
			LH (ng/ml)		
No.	A	B	C	D	E
50942	0.03	0.06	0.07	0.05	0.12
50618	0.09	0.09	0.06	0.09	0.12
40546	0.09	0.09	0.06	0.08	0.07
50606	0.06	0.04	0.07	0.11	0.09
40548	0.03	0.06	0.06	0.1	0.08
50632	0.04	0.03	0.06	0.13	0.15
50622	0.04	0.14	0.09	0.09	0.05
50609	0.03	0.04	0.07	0.05	0.06
45636	0.06	0.05	0.08	0.07	0.1

47894	0.12	0.06	0.08	0.09	0.1
50287	0.04	0.09	0.06	0.09	0.11
48995	0.09	0.04	0.08	0.08	0.1
56055	0.08	0.1	0.03	0.07	0.11
52990	0.08	0.1	0.08	0.09	0.11
57058	0.06	0.14	0.08	0.09	0.09
48095	0.05	0.05	0.08	0.1	0.07
58438	0.08	0.04	0.09	0.13	0.11
47637	0.06	0.05	0.09	0.14	0.07
55915	0.03	0.11	0.08	0.07	0.04
51461	0.07	0.05	0.06	0.14	0.07
42758	0.04	0.1	0.06	0.04	0.09
58067	0.02	0.07	0.08	0.03	0.09
40567	0.04	0.05	0.08	0.04	0.11
54080	0.11	0.06	0.08	0.07	0.1
52864	0.06	0.08	0.04	0.09	0.12
Mean	0.060	0.071	0.070	0.085	0.093
SD	0.026	0.030	0.014	0.030	0.025
P	A	AB	AB	BC	C
Mean±SD	0.06±0.026	0.071±0.03	0.07±0.014	0.085±0.03	0.093±0.025

Table S3. Qualitative results of differential metabolites.

HMDB_ID	Description	m/z	Retention time (min)	UP or DOWN	
HMDB0000157	Hypoxanthine	137.0457	3.181367	B/A	DOWN
				D/B	DOWN
HMDB0000195	Inosine	291.0696	4.3967	D/B	DOWN
HMDB0000205	2-Oxo-3-phenylpropanoic acid (Mixture oxo and keto)	165.0546	6.00885	E/D	UP
HMDB0000477	7Z,10Z-Hexadecadienoic acid	297.207	6.766433	E/A	DOWN
HMDB0000552	3-Methylglutarylcarnitine	290.1594	5.221317	E/A	DOWN
HMDB0000759	Glycylleucine	189.1234	1.53895	D/A	UP
				E/A	UP
HMDB0000942	Tetrahydroneopterin	258.1193	3.248	B/A	DOWN
				E/A	UP
HMDB0000949	Tetrahydrocortisol	367.2472	7.235	D/B	UP
HMDB0000969	1,25-Dihydroxyvitamin D3-26,23-lactone	445.2942	9.014483	C/B	DOWN
HMDB0001049	Gamma-Glutamylcysteine	295.0606	5.5979	E/B	UP
HMDB0001125	Inositol cyclic phosphate	287.0166	5.014633	E/A	UP
HMDB0001867	4-Aminohippuric acid	195.0764	5.188733	C/A	DOWN
HMDB0001896	5-Methoxytryptophol	192.1018	6.608033	E/C	DOWN
HMDB0001929	Levofloxacin	362.1526	1.41285	E/A	DOWN
HMDB0002689	13,14-Dihydro PGE1	357.263	6.837917	C/A	UP
HMDB0003040	Arabinosylhypoxanthine	267.0734	4.40185	D/A	DOWN
				D/B	DOWN
HMDB0003339	D-Glutamic acid	148.0602	7.069733	D/B	UP
HMDB0004049	20-Hydroxy-PGF2a	415.234	6.696883	D/B	UP
HMDB0004198	Reduced Vitamin K (phylloquinone)	449.3425	10.68177	E/A	DOWN
HMDB0004667	13S-hydroxyoctadecadienoic acid	341.2334	7.452883	D/A	DOWN
HMDB0005821	Beta-Cortol	369.2627	7.36205	E/A	UP
HMDB0006045	Dityrosine	361.139	5.788133	C/A	UP
HMDB0006372	L-Glyceric acid	151.0249	3.89215	D/B	UP
HMDB0006469	Linoleyl carnitine	424.3413	6.948333	B/A	DOWN
HMDB0006940	9(S)-HPODE	357.2282	7.299417	D/A	DOWN
				D/B	DOWN
HMDB0007931	PC(14:1(9Z)/P-18:1(9Z))	712.5291	13.15612	E/C	DOWN
HMDB0007961	PC(15:0/P-16:0)	702.5448	13.88853	C/A	UP
				E/B	DOWN
				E/C	DOWN
HMDB0008064	PC(18:1(11Z)/14:0)	730.54	13.63198	D/C	DOWN
				E/A	DOWN
				E/B	DOWN
				E/C	DOWN
HMDB0008394	PC(20:3(8Z,11Z,14Z)/14:0)	754.5395	13.15612	D/C	DOWN
				E/C	DOWN
HMDB0008850	PE(14:0/P-16:0)	646.4823	11.96288	C/A	UP
HMDB0008892	PE(15:0/18:0)	704.5241	13.16865	E/A	DOWN
				E/C	DOWN
				E/D	DOWN
HMDB0008916	PE(15:0/P-16:0)	706.5183	12.73318	E/C	DOWN
HMDB0009048	PE(18:1(11Z)/P-16:0)	700.5291	13.60697	D/C	DOWN
				E/C	DOWN
HMDB0009378	PE(20:3(8Z,11Z,14Z)/P-16:0)	724.5291	13.25757	E/C	DOWN

HMDB_ID	Description	m/z	Retention time (min)	UP or DOWN	
HMDB0010166	PS(18:0/22:5(7Z,10Z,13Z,16Z,19Z))	838.5559	9.232483	C/A	UP
				E/A	UP
HMDB0010316	Acetaminophen glucuronide	326.0882	5.619233	D/B	UP
HMDB0010569	PE-NMe(16:0/18:1(9Z))	730.5396	13.3909	E/B	DOWN
				E/C	DOWN
HMDB0010730	3-Oxotetradecanoic acid	241.1806	7.517267	B/A	UP
HMDB0011386	PE(P-18:0/20:4(8Z,11Z,14Z,17Z))	750.5448	13.94273	E/C	DOWN
HMDB0011401	PE(P-18:1(11Z)/14:0)	672.4979	12.35693	E/C	DOWN
HMDB0011403	PE(P-18:1(11Z)/15:0)	686.5132	12.74393	E/C	DOWN
HMDB0011416	PE(P-18:1(11Z)/20:3(5Z,8Z,11Z))	750.5444	13.40813	E/C	DOWN
HMDB0011760	Cer(d18:0/16:0)	584.5262	12.75997	D/C	DOWN
HMDB0011761	Cer(d18:0/18:0)	612.5578	13.82073	D/A	DOWN
				D/B	DOWN
				D/C	DOWN
				E/A	DOWN
				E/B	DOWN
				E/C	DOWN
HMDB0011763	Cer(d18:0/18:1(9Z))	610.5417	13.26915	D/C	DOWN
				E/C	DOWN
HMDB0013208	9-Hexadecenoylcholine	363.3096	8.3496	D/C	DOWN
HMDB0013246	Margaroylglycine	372.2753	7.47975	E/A	DOWN
HMDB0013331	3, 5-Tetradecadiencarnitine	368.2791	6.624133	D/A	DOWN
HMDB0013648	Palmitoleoyl Ethanolamide	298.2735	7.889533	D/C	DOWN
				D/A	DOWN
HMDB0013973	5-Hydroxymethyl tolterodine	386.2337	8.337483	C/A	DOWN
HMDB0014353	Ticlopidine	308.0519	1.385783	E/B	DOWN
HMDB0014727	Lisuride	361.2002	7.149583	D/A	UP
				D/B	UP
HMDB0015086	Isoniazid	138.0661	3.120233	E/A	DOWN
HMDB0015133	Frovatriptan	244.1442	5.852083	D/A	UP
				E/A	UP
HMDB0015330	Tubocurarine	632.2873	5.022317	D/A	DOWN
				D/B	DOWN
HMDB0015377	Isocarboxazid	232.1079	4.89115	D/A	DOWN
				E/A	UP
HMDB0015668	Gestodene	355.1917	8.654433	B/A	DOWN
				C/A	DOWN
HMDB0028716	Arginyl-Phenylalanine	322.1871	4.41275	C/A	UP
HMDB0029081	Tryptophyl-Glutamine	377.1454	6.04845	C/A	DOWN
				D/A	DOWN
				E/D	UP
HMDB0029112	Tyrosyl-Phenylalanine	373.1403	6.138767	E/A	DOWN
HMDB0029638	4-Methylbenzaldehyde	121.0648	6.953567	D/C	DOWN
HMDB0029838	Harmalol	199.087	5.10765	C/A	DOWN
HMDB0030254	propanoic acid	241.1067	7.149583	E/A	DOWN
				E/C	DOWN
				E/D	DOWN
HMDB0030492	Spirolide B	694.4645	8.854283	C/A	UP
HMDB0030686	Cyclointegrin	369.1331	7.2136	C/B	DOWN
HMDB0030903	3-(4-Methylphenyl)oxiranecarboxylic acid	223.0605	6.208817	B/C	DOWN
				C/A	UP

HMDB_ID	Description	m/z	Retention time (min)	UP or DOWN	
				D/A	UP
				E/A	UP
HMDB0031048	Avocadyne 1-acetate	371.2437	7.373383	D/B	UP
HMDB0031054	10-Hydroxy-2,8-decadiene-4,6-diynoic acid	177.0545	6.693783	D/B	DOWN
HMDB0031486	3-Hepten-2-one	113.0964	5.027767	D/A	DOWN
				E/A	DOWN
HMDB0031929	Zanthodioline	304.1191	6.50105	C/A	UP
HMDB0031972	Heliespirone A	265.1432	6.7421	E/A	UP
HMDB0032240	N-3,7-Dimethyl-2,6-octadienylcyclopropylcarboxamide	222.1851	6.019817	E/A	DOWN
HMDB0032662	(S)-9-Hydroxy-10-undecenoic acid	401.2891	7.36205	D/B	UP
HMDB0032864	Mycotoxin T 2	342.1541	5.809383	E/A	DOWN
HMDB0033123	4-Phenylpyridine	156.0807	5.0441	D/B	UP
				E/B	UP
HMDB0033433	(S)-Homostachydrine	180.0991	2.297217	B/A	DOWN
				C/A	DOWN
				D/A	DOWN
				E/A	DOWN
HMDB0033512	Gonyautoxin VI	394.0779	4.753167	E/A	UP
				E/B	UP
				E/C	UP
HMDB0033643	10-Hydroxy-8-nor-2-fenchanone glucoside	361.1507	6.686133	D/A	DOWN
HMDB0034056	5-(3,4-Methylenedioxyphenyl)pentanoic acid	223.0964	5.509367	D/A	DOWN
HMDB0034206	Ethyl 4-methylphenoxyacetate	239.0922	7.171183	C/A	UP
HMDB0034276	L,L-Cyclo(leucylprolyl)	255.1348	5.923983	D/A	UP
				E/A	UP
HMDB0034293	Asperagenin	447.3113	8.166317	E/A	UP
HMDB0034672	(5alpha,8beta,9beta)-5,9-Epoxy-3,6-megastigmadien-8-ol	417.2992	8.009933	E/C	UP
HMDB0034881	3-Carboxy-2,3,4,9-tetrahydro-1H-pyrido[3,4-b]indole-1-propanoic acid	289.118	5.04955	B/A	DOWN
				E/A	UP
HMDB0035404	Isogingerenone B	387.1795	6.953567	E/C	DOWN
HMDB0035571	4,6-Heneicosanedione	325.3096	9.349667	E/A	UP
HMDB0035779	Armillane	421.2214	7.235	E/A	DOWN
				E/C	DOWN
				E/D	DOWN
HMDB0036006	Xanthopurpurin	285.0403	4.811733	D/B	UP
				E/B	DOWN
HMDB0036021	Rhubafuran	221.1178	6.936767	C/A	DOWN
				C/B	DOWN
				E/C	UP
HMDB0036031	Labienoxime	254.176	7.522583	B/A	DOWN
HMDB0036093	Abscisic acid	263.1288	7.144533	B/A	DOWN
HMDB0036195	N,2,3-Trimethyl-2-(1-methylethyl)butanamide	343.3312	7.615833	D/A	DOWN
HMDB0036731	beta-Neoclovene	205.1949	7.069733	C/B	DOWN
				E/C	UP
HMDB0036756	(ent-6alpha,7alpha,16alphaH)-6,7,17-Trihydroxy-19-kauranoic acid	397.2231	8.027517	D/A	UP
				D/B	UP
				E/D	UP
HMDB0037028	Deoxyloganic acid	383.1309	5.67545	C/A	DOWN
				C/B	DOWN
				D/A	DOWN
				E/C	UP

HMDB_ID	Description	m/z	Retention time (min)	UP or DOWN	
HMDB0037519	2-(5,8-Tetradecadienyl)cyclobutanone	263.2363	8.31305	D/A	DOWN
				E/A	UP
HMDB0037834	Ethyl menthane carboxamide	256.1918	7.7502	E/B	DOWN
				E/D	DOWN
HMDB0038057	Dehydrophytosphingosine	316.284	7.910517	B/A	DOWN
				E/A	DOWN
HMDB0038128	Sphaerosin	605.2361	4.697667	E/C	DOWN
HMDB0038522	2-(10-Heptadecenyl)-6-hydroxybenzoic acid	419.2802	8.038183	D/A	DOWN
HMDB0038523	Methyl 2-(10-heptadecenyl)-6-hydroxybenzoate	433.2961	8.048783	E/C	UP
HMDB0038630	Niazimin A	382.1507	5.983567	E/D	UP
HMDB0038794	2,12-Epoxy-7(14)-illudadiene-3,8-diol	249.1482	5.719267	E/C	DOWN
				E/D	DOWN
				C/A	DOWN
				C/B	DOWN
HMDB0039155	ar-Artemisene	271.2413	7.884267	D/A	DOWN
				E/A	DOWN
				E/B	DOWN
HMDB0040033	2-Heptylbenzothiazole	467.2544	4.769417	E/B	DOWN
				E/D	DOWN
				B/A	DOWN
HMDB0040045	5-[2H-Pyrrol-4-(3H)-ylidenemethyl]-2-furanmethanol	222.0766	5.102183	D/A	UP
				E/A	UP
HMDB0040694	Agaritinal	266.1134	5.32935	C/A	UP
HMDB0040768	N-(2,5-Dihydroxyphenyl)pyridinium(1+)	377.1508	4.42895	E/C	DOWN
HMDB0040972	1-Methoxy-1H-indole-3-carboxaldehyde	176.0705	4.3062	E/A	DOWN
HMDB0041787	Urolithin B 3-O-glucuronide	389.086	6.203733	E/B	UP
HMDB0043117	TG(15:0/22:0/22:1(13Z))	957.8833	4.78875	C/A	UP
				C/A	UP
HMDB0059631	Cis-stilbene oxide	197.0958	7.34645	E/B	DOWN
				E/C	DOWN
				E/D	DOWM
HMDB0059856	Ethyl 2-pyrrolecarboxylate	279.1336	6.08525	D/B	DOWN
				D/C	DOWN
HMDB0060107	(5Z,9E,12S,14Z)-8,11,12-Trihydroxyicosa-5,9,14-trienoate	355.2472	7.36205	C/B	DOWN
				E/B	UP
HMDB0060484	Indolepyruvate	248.0561	5.382483	E/A	UP
HMDB0060747	3-O-Methyl-a-methyldopa	212.0916	5.997917	C/A	UP
HMDB0060776	5-Hydroxydantrolene	329.0515	5.20685	D/B	UP

A. B, C, D and E represent the grouping of 5 time points; DOWN indicates that the expression of metabolites is down regulated in the group represented by molecules compared with the group represented by denominator; Similarly, UP expression was up-regulated. For example, "E/A, DOWN" indicates that the expression of this metabolite is down regulated in group E compared with group A.

Table S4. The result of KEGG analysis.

No.	category	annotation	Count
1	Cellular Processes	Cell growth and death	HMDB0001049
2		Transport and catabolism	HMDB0009378
3	Drug Development	Target-based classification: Ion channels	HMDB0015330
4	Environmental Information Processing	Membrane transport	HMDB0000195
5	Human Diseases	Cancer: overview	HMDB0007961
6		Immune disease	HMDB0010166
7		Infectious disease: bacterial	HMDB0009378
8		Infectious disease: parasitic	HMDB0010166
9		Infectious disease: viral	HMDB0009378
10		Neurodegenerative disease	HMDB0033123
11	Metabolism	Lipid metabolism	HMDB0006940<br>HMDB0007961<br>HMDB0009378<br>HMDB0004667<br>HMDB0000949<br>HMDB0010166<br>HMDB0060484
12		Amino acid metabolism	HMDB0000205<br>HMDB0010166
13		Chemical structure transformation maps	HMDB0000205<br>HMDB0015330
14		Metabolism of other amino acids	HMDB0001049<br>HMDB0003339
15		Nucleotide metabolism	HMDB0000195<br>HMDB0000157
16		Xenobiotics biodegradation and metabolism	HMDB0029638<br>HMDB0015086
17		Biosynthesis of other secondary metabolites	HMDB0000205
18		Glycan biosynthesis and metabolism	HMDB0009378
19	Organismal Systems	Digestive system	HMDB0033123<br>HMDB0001929
20		Nervous system	HMDB0007961<br>HMDB0009378
21		Endocrine system	HMDB0004667

Table S5. The result of HMDB analysis.

No.	annotation	Count
1	Lipids and lipid-like molecules	HMDB0000477; HMDB0000552; HMDB0000949; HMDB0000969; HMDB0002689; HMDB0004049; HMDB0004198; HMDB0004667; HMDB0005821; HMDB0006469; HMDB0006940; HMDB0007931; HMDB0007961; HMDB0008394; HMDB0008850; HMDB0008892; HMDB0008916; HMDB0009048; HMDB0009378; HMDB0010166; HMDB0010569; HMDB0010730; HMDB0011386; HMDB0011401; HMDB0011403; HMDB0011416; HMDB0011760; HMDB0011761; HMDB0011763; HMDB0015668; HMDB0031048; HMDB0031054; HMDB0032240; HMDB0033643; HMDB0034293; HMDB0035779; HMDB0036093; HMDB0036195; HMDB0036731; HMDB0036756; HMDB0037028; HMDB0037834; HMDB0039155; HMDB0043117; HMDB0060107
2	Organoheterocyclic compounds	HMDB0000157; HMDB0000942; HMDB0001896; HMDB0001929; HMDB0014353; HMDB0014727; HMDB0015086; HMDB0015133; HMDB0030492; HMDB0030686; HMDB0031929; HMDB0033123; HMDB0034056; HMDB0034672; HMDB0040033; HMDB0040045; HMDB0040972; HMDB0059856; HMDB0060484; HMDB0060776
3	Organic acids and derivatives	HMDB0000759; HMDB0001049; HMDB0001125; HMDB0003339; HMDB0013246; HMDB0013331; HMDB0028716; HMDB0029081; HMDB0029112; HMDB0032662; HMDB0033433; HMDB0034276; HMDB0040694; HMDB0060747
4	Benzenoids	HMDB0000205; HMDB0001867; HMDB0013973; HMDB0015377; HMDB0029638; HMDB0030903; HMDB0034206; HMDB0036006; HMDB0036021; HMDB0038522; HMDB0038523; HMDB0040768
5	Organic oxygen compounds	HMDB0006372; HMDB0010316; HMDB0015330; HMDB0031486; HMDB0031972; HMDB0032864; HMDB0035571; HMDB0038630; HMDB0041787
6	Phenylpropanoids and polyketides	HMDB0030254; HMDB0033512; HMDB0035404; HMDB0038128; HMDB0059631
7	Organic nitrogen compounds	HMDB0013208; HMDB0013648; HMDB0036031; HMDB0038057
8	Alkaloids and derivatives	HMDB0029838; HMDB0034881
9	Nucleosides, nucleotides, and analogues	HMDB0000195; HMDB0003040
10	Organooxygen compounds	HMDB0037519
11	Lignans, neolignans and related compounds	HMDB0006045

Table S6. The result of LMSD analysis.

No.	category	annotation	Count
1	Sterol Lipids [ST]	Steroids [ST02]	HMDB0001049
2		Secosteroids [ST03]	HMDB0009378
3	Sphingolipids [SP]	Ceramides [SP02]	HMDB0015330
4	Polyketides [PK]	Flavonoids [PK12]	HMDB0000195
5		Aromatic polyketides [PK13]	HMDB0007961
6	Glycerophospholipids [GP]	Glycerophosphocholines [GP01]	HMDB0010166
7		Glycerophosphoethanolamines [GP02]	HMDB0009378
8	Fatty Acyls [FA]	Fatty Acids and Conjugates [FA01]	HMDB0010166
9		Fatty esters [FA07]	HMDB0009378
10		Fatty amides [FA08]	HMDB0033123
11		Eicosanoids [FA03]	HMDB0006940; HMDB0007961 HMDB0009378; HMDB0004667 HMDB0000949; HMDB0010166
12		Fatty alcohols [FA05]	HMDB0060484; HMDB0000205 HMDB0010166
13		Oxygenated hydrocarbons [FA12]	HMDB0000205; HMDB0015330
